# Supplementary material for: HIVprotI: an integrated web based platform for prediction and design of HIV proteins inhibitors
Source: J Cheminform. 2018 Mar 9;10:12. doi: 10.1186/s13321-018-0266-y (PMC5845081; doi:10.1186/s13321-018-0266-y)
Supplement: Supplementary file 2 — Additional file 2. Source code of HIVProtI web server. [file 13321_2018_266_MOESM2_ESM.zip › HIVprotI_Source-code/hivprotI/weka-3-6-6/doc/weka/core/AllJavadoc.html]

AllJavadoc


---


|  |  |  |
| --- | --- | --- |
| **Overview** \| **Package** \| **Class** \| **Tree** \| **Deprecated** \| **Index** \| **Help** \| **Weka's home** | |  |
| **PREV CLASS**   **NEXT CLASS** | **FRAMES**    **NO FRAMES**     **All Classes** |  |
| SUMMARY: NESTED \| FIELD \| CONSTR \| METHOD | DETAIL: FIELD \| CONSTR \| METHOD |  |


---


## weka.core Class AllJavadoc

```
java.lang.Object
  weka.core.Javadoc
      weka.core.AllJavadoc
```

**All Implemented Interfaces:**: OptionHandler, RevisionHandler

---

``` public class AllJavadoc extends Javadoc ```

Applies all known Javadoc-derived classes to a source file.
Valid options are:

```
 -W <classname>
  The class to load.
```

```
 -nostars
  Suppresses the '*' in the Javadoc.
```

```
 -dir <dir>
  The directory above the package hierarchy of the class.
```

```
 -silent
  Suppresses printing in the console.
```

**Version:**
:   $Revision: 6110 $

**Author:**
:   fracpete (fracpete at waikato dot ac dot nz)

---

**Constructor Summary**

`AllJavadoc()`


| **Method Summary** | |
| --- | --- |
| `java.lang.String` | `getRevision()`             Returns the revision string. |
| `static void` | `main(java.lang.String[] args)`             Parses the given commandline parameters and generates the Javadoc. |
| `void` | `setClassname(java.lang.String value)`             sets the classname of the class to generate the Javadoc for |
| `void` | `setSilent(boolean value)`             sets whether to suppress output in the console |
| `void` | `setUseStars(boolean value)`             sets whether to prefix the Javadoc with "\*" |

**Methods inherited from class weka.core.Javadoc**

`generate, generateHelp, getClassname, getDir, getOptions, getSilent, getUseStars, listOptions, setDir, setOptions, updateJavadoc`

**Methods inherited from class java.lang.Object**

`equals, getClass, hashCode, notify, notifyAll, toString, wait, wait, wait`

**Constructor Detail**

### AllJavadoc

```
public AllJavadoc()
```


**Method Detail**

### setClassname

```
public void setClassname(java.lang.String value)
```

:   sets the classname of the class to generate the Javadoc for

    :   **Overrides:**: `setClassname` in class `Javadoc`
    :   **Parameters:**: `value` - the new classname

---


### setUseStars

```
public void setUseStars(boolean value)
```

:   sets whether to prefix the Javadoc with "\*"

    :   **Overrides:**: `setUseStars` in class `Javadoc`
    :   **Parameters:**: `value` - true if stars are to be used

---


### setSilent

```
public void setSilent(boolean value)
```

:   sets whether to suppress output in the console

    :   **Overrides:**: `setSilent` in class `Javadoc`
    :   **Parameters:**: `value` - true if output is to be suppressed

---


### getRevision

```
public java.lang.String getRevision()
```

:   Returns the revision string.

    :   **Returns:**: the revision

---


### main

```
public static void main(java.lang.String[] args)
```

:   Parses the given commandline parameters and generates the Javadoc.

    :   **Parameters:**: `args` - the commandline parameters for the object


---


|  |  |  |
| --- | --- | --- |
| **Overview** \| **Package** \| **Class** \| **Tree** \| **Deprecated** \| **Index** \| **Help** \| **Weka's home** | |  |
| **PREV CLASS**   **NEXT CLASS** | **FRAMES**    **NO FRAMES**     **All Classes** |  |
| SUMMARY: NESTED \| FIELD \| CONSTR \| METHOD | DETAIL: FIELD \| CONSTR \| METHOD |  |


---
